# Redefining regeneration: proposal of a radiographic outcome-based framework for evaluating regenerative endodontic procedures in immature necrotic teeth

**DOI:** 10.2340/biid.v13.46956

**Published:** 2026-10-08

**Authors:** Ruchi Singhal, Akshita Bansal, Shiwani Chawla, Ritu Namdev

**Affiliations:** Department of Pediatric and Preventive Dentistry, Post Graduate Institute of Dental Sciences, Rohtak, Haryana, India

**Keywords:** Regenerative endodontics, revascularization, immature teeth, open apex, radiographic classification

## Abstract

**Purpose:**

To propose a radiographic classification system for evaluating outcomes of regenerative endodontic procedures (REPs) in immature necrotic permanent teeth.

**Methods:**

A two-phase retrospective study was conducted. In Phase I, radiographs of 20 cases (2015–2018) were analyzed to identify patterns of apical closure, root lengthening, dentinal wall thickening, intracanal calcification, and periapical healing. Based on these findings, a six-class radiographic classification was proposed:

Class 1: Apical closure with increased root length and dentin thickness;

Class 2: Apical closure with increased root length only;

Class 3: Apical closure with increased dentin thickness only;

Class 4: Apical closure without changes in root length or dentin thickness;

Class 5: Barrier formation within the canal or coronal to the root end;

Class 6: No root dimensional changes with periapical healing of a documented pretreatment periapical radiolucency.

In Phase II, the classification was applied to 54 immature single-rooted permanent teeth from 44 patients aged 8–13 years treated with REPs between 2019 and 2023. Baseline and latest available follow-up radiographs were evaluated by two blinded examiners. Reproducibility was assessed using Cohen’s kappa, and associations with age and pre-treatment root stage were analyzed using Fisher’s exact test.

**Results:**

Class 1 was most frequent. Partial intracanal calcification occurred in 35.18% of cases, with no complete canal obliteration. Age (*p* = 0.215) and pre-treatment root stage (*p* = 0.872) were not significantly associated with outcome classes. Inter- and intra-examiner agreement were high (κ = 0.82 and 0.86). All cases were accommodated within the six proposed categories, and no additional outcome classes were observed.

**Conclusion:**

This proposed six-class radiographic framework demonstrated good internal reproducibility and may provide a structured basis for outcome reporting after REPs. Prospective multicenter validation is required.

## Introduction

The management of immature permanent teeth with pulp necrosis and/or periapical lesions remains one of the most challenging aspects of endodontic therapy. Traditionally, apexification using long-term calcium hydroxide application was employed to induce apical closure [1, 2]. Alternatively, single-step apexification using mineral trioxide aggregate (MTA) was introduced to create an artificial apical barrier [3, 4]. However, neither of these techniques promotes continued root development or thickening of the radicular dentin. Consequently, treated teeth remain prone to root fractures, complicating long-term outcomes and prognosis [5, 6]. These limitations prompted the development of regenerative endodontics – a biologically based approach rooted in tissue engineering principles. This method aims to replace damaged pulp tissue with viable cells from the pulp-dentin complex, fostering continued root development (maturogenesis) and increasing dentinal wall thickness, thereby enhancing the structural integrity of the affected tooth [7–9]. In the early 2000s, regenerative endodontic procedures (REPs) were explored for treating immature necrotic teeth, with case reports and series demonstrating favorable outcomes [10–12]. Later studies comparing REPs with conventional apexification showed similar resolution of periapical pathology but greater increases in root length and dentin wall thickness with REPs [13–15].

Standardized treatment protocols and follow-up evaluation criteria have since been provided by the American Association of Endodontists [16] and the European Society of Endodontology [17]. The American Association of Endodontists (AAE) categorizes the success of REPs into three hierarchical goals. The primary goal is the elimination of symptoms and bony healing. The secondary goal is the increased root wall thickness and/or root length, which is desirable but not essential. The tertiary goal is a positive response to vitality testing, which may indicate the presence of more organized, vital pulp tissue. Recent systematic reviews suggest that the primary goal is reliably achieved [18, 19]. However, although previous studies have evaluated radiographic changes in parameters such as apical diameter, root length, dentinal-wall thickness, and radiographic root area following REPs, these parameters have generally been assessed separately rather than integrated into a component-based outcome classification [20–25]. Chen et al. [22] described several radiographic response patterns following revascularization, which were subsequently adapted by Shivashankar et al. [23] and Thakkar et al. [24]. However, these classifications primarily described observed patterns rather than providing an objective, component-based approach to distinguish individual features of root maturation.

The present study therefore proposes and internally applies a component-based modification and extension of these concepts, assessing root maturation through three principal radiographic components: apical maturation/closure, continued root lengthening, and dentinal-wall thickening. This framework is intended to improve the objectivity and reproducibility of radiographic outcome assessment and facilitate more consistent evaluation of REPs.

## Methods

### Study design and setting

The proposed classification system to categorize the outcomes of REPs was developed using a two-phase retrospective design.

**Phase I** involved the development of a radiographic outcome classification framework, wherein a pilot analysis of 20 cases treated between 2015 and 2018 was undertaken to identify patterns of radiographic healing following REPs and to formulate preliminary classification criteria.

**Phase II** involved the application and evaluation of the proposed classification in an independent cohort of cases treated between 2019 and 2023, forming the basis of the present analysis.

The study was conducted in the Department of Pedodontics and Preventive Dentistry and evaluated the clinical and radiographic records of children who had undergone REPs. The study adhered to the Strengthening the Reporting of Observational Studies in Epidemiology (STROBE) guidelines [26]. Ethical approval for the study was obtained from the Institutional Ethics Committee. Written informed consent had been obtained from all patients prior to initiation of treatment and in the event of any modification to the treatment plan. The largely standardized clinical protocol, data collection, and measurement methods were identical for both phases.

### Participants

Cases for both Phase I and Phase II were selected using the same predefined inclusion and exclusion criteria.

### Inclusion criteria

- Patients younger than 14 years at the time of treatment
- Single-rooted immature permanent teeth diagnosed with pulp necrosis, with or without periapical pathology, due to trauma or caries
- In trauma-related cases, only dental hard-tissue injuries were included, excluding soft-tissue, periodontal, or luxation injuries
- Cases treated using largely standardized regenerative endodontic protocols
- Availability of complete clinical and radiographic records with a minimum follow-up period of 2 years
- Medically and systemically healthy individuals

### Exclusion criteria

- Teeth treated using conventional apexification techniques (e.g. calcium hydroxide or MTA/Biodentine apical plug)
- Follow-up period of less than 2 years
- Incomplete clinical or radiographic documentation
- Deviations from the largely standardized treatment protocol (e.g. changes in scaffold, intracanal medicament, or irrigants)
- Retreatment cases of REPs.

### Clinical protocol

The clinical procedure followed the protocol outlined in the AAE guidelines, ‘Clinical Considerations for a Regenerative Procedure’ [16]. After access cavity preparation under rubber dam isolation, canals were gently irrigated with 1.5% sodium hypochlorite (20 mL per canal over 5 min) and subsequently flushed with sterile saline (20 mL per canal over 5 min). Triple antibiotic paste (TAP), consisting of ciprofloxacin, metronidazole, and minocycline in a 1:1:1 ratio, with each antibiotic at a final concentration of 1 mg/mL, was prepared by mixing the powdered antibiotics with sterile saline and then placed intracanal using a Lentulo spiral. The paste was confined below the cemento-enamel junction, followed by placement of a temporary restorative material.

At the second visit (1–4 weeks later), if clinical symptoms had resolved, the canal was irrigated with 20 mL of 17% ethylenediaminetetraacetic acid (EDTA). After drying, bleeding was induced using a pre-curved K-file positioned 2 mm beyond the apex, aiming to fill the canal up to the cemento-enamel junction or mid-root level. Cases in which adequate bleeding could not be induced and alternative scaffolds (e.g. platelet-rich plasma [PRP] or platelet-rich fibrin) were used were excluded from analysis to minimize variability.

A calcium silicate–based material – either MTA (ProRoot MTA, Dentsply Sirona, USA) or Biodentine™ (Septodont, France) – was placed over the stabilized blood clot depending on material availability. This was followed by placement of a 3–4 mm layer of resin-modified glass ionomer liner (Vitrebond™, 3M ESPE, Germany) before final restoration. Follow-up evaluations were scheduled at 6, 12, and 24 months.

### Data collection

Data collected included demographic information, clinical findings, and radiographic assessments. All radiographic assessments were performed using conventional intraoral periapical radiographs acquired using the paralleling technique as part of routine clinical care. The examiners were trained and calibrated before assessment. Examiner calibration refers to observer training and agreement on classification criteria and should be distinguished from radiographic image calibration. No dedicated software-based image registration or dimensional calibration was performed, and correction for radiographic projection-related magnification was not consistently available in this retrospective dataset.

Recorded variables comprised tooth type and number, stage of root development (using Cvek’s classification), apical diameter, root length, dentin thickness, presence or absence of periapical radiolucency, etiology (caries or trauma), treatment-related variables (irrigation protocol, intracanal medicament, scaffold type, bleeding status, coronal seal material), and duration of follow-up. Preoperative root development was classified according to Cvek’s staging criteria [27] (Stage 1: Less than half root length, Stage 2: Half root length, Stage 3: Up to two-thirds root length, Stage 4: Wide open apical foramen and nearly complete root length, and Stage 5: Closed apical foramen and completed root development), using the adjacent tooth as the developmental reference. The latest available follow-up radiograph for each tooth was used for final classification and analysis. The minimum follow-up period was 24 months.

### Outcome evaluation

Treatment outcomes were assessed clinically and radiographically. Because the objective was to categorize secondary radiographic responses after a favorable primary outcome (as defined by AAE), classification was restricted to teeth that were clinically asymptomatic and showed no persistent or worsening periapical disease. The criteria for assessment were taken from a previous study by Bose et al. [28]. Root length was defined as the linear distance from a reproducible cemento-enamel junction (CEJ) reference point to the radiographic root apex along the long axis of the tooth. Apical diameter was defined as the width of the radiographically visible canal opening at the anatomical root end (Figure 1); apical closure was recorded when no radiographically discernible communication remained between the canal lumen and the periapical tissues. Root dentin thickness was measured at a fixed distance of 6.5 mm apical to the CEJ and calculated as (total external root width – canal width)/2 at a line perpendicular to the long axis. This 6.5 mm level was selected because the average root length of a fully developed maxillary central incisor is approximately 13 mm, making 6.5 mm representative of the mid-root region. The use of a reproducible CEJ-based reference also facilitated standardized assessment among immature teeth at different stages of root development. For teeth with a pretreatment radiolucency, complete periapical healing was defined as disappearance of the radiolucency with re-establishment of a normal or near-normal trabecular bone pattern and periodontal-ligament outline. Continued healing was defined as a clear reduction in radiolucency accompanied by progressive trabecular bone fill, without symptoms, new pathology, or radiographic progression. Teeth without pretreatment pathology could demonstrate maintained periapical health but were not described as showing healing. Because this was a retrospective radiographic classification study, no prespecified quantitative threshold for change in root length, dentin thickness, or reduction in periapical radiolucency was applied; classification was based on comparative radiographic assessment.

**Figure 1:**
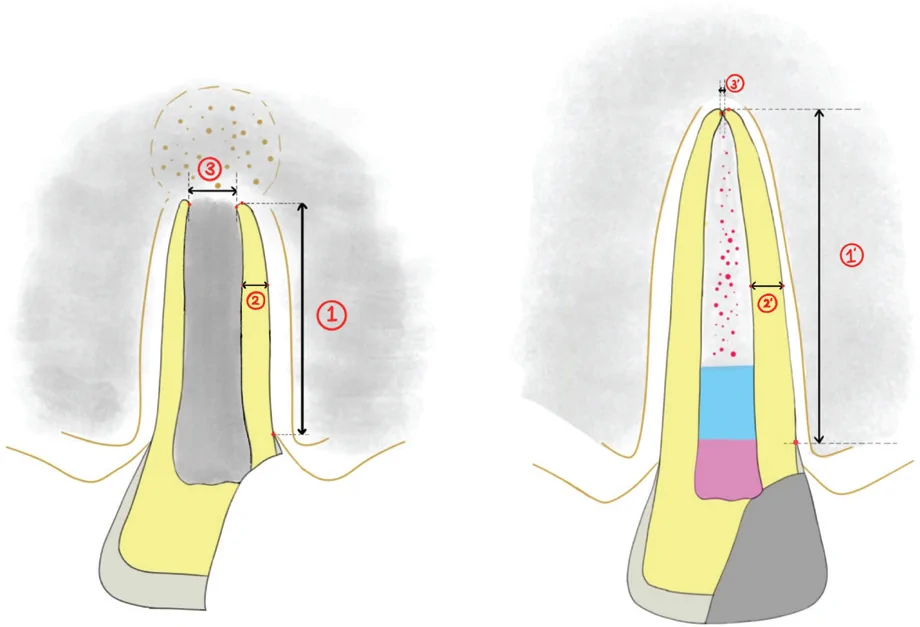
Illustrations depicting pre- and post-treatment images showing three assessed parameters: 1 and 1’- root length measured from CEJ to root apex, 2 and 2’-root dentin wall thickness measured at 6.5 mm apical to CEJ, 3 and 3’- apical diameter.

In Phase I, these radiographic parameters were used to identify recurring patterns that guided development of the classification framework. In Phase II, the same parameters were applied to assign cases to predefined categories and assess reproducibility**.** The framework describes radiographic outcomes and should not be interpreted as a treatment-guiding decision algorithm.

### Phase I: Proposal of classification system

Radiographic outcomes from the pilot cohort of 20 patients treated between 2015 and 2018 were analyzed to identify consistent patterns of apical closure, root lengthening, dentinal wall thickening, intracanal calcification, and periapical healing, which formed the basis of the proposed classification framework and were assessed using both preoperative and postoperative radiographs.

## Radiographic outcome classification framework

Based on the radiographic patterns observed during Phase I, a proposed modified classification system – hereafter referred to as the **Singhal and Bansal classification system** – was formulated to categorize outcomes following REPs. To improve clarity and objectivity, the principal class was determined from three components of root maturation: apical maturation/closure, root lengthening, and dentinal-wall thickening. This framework stratifies the progression and maturation of root development into six distinct categories to facilitate standardized outcome assessment and inter-study comparisons (Figure 2):

**Figure 2:**
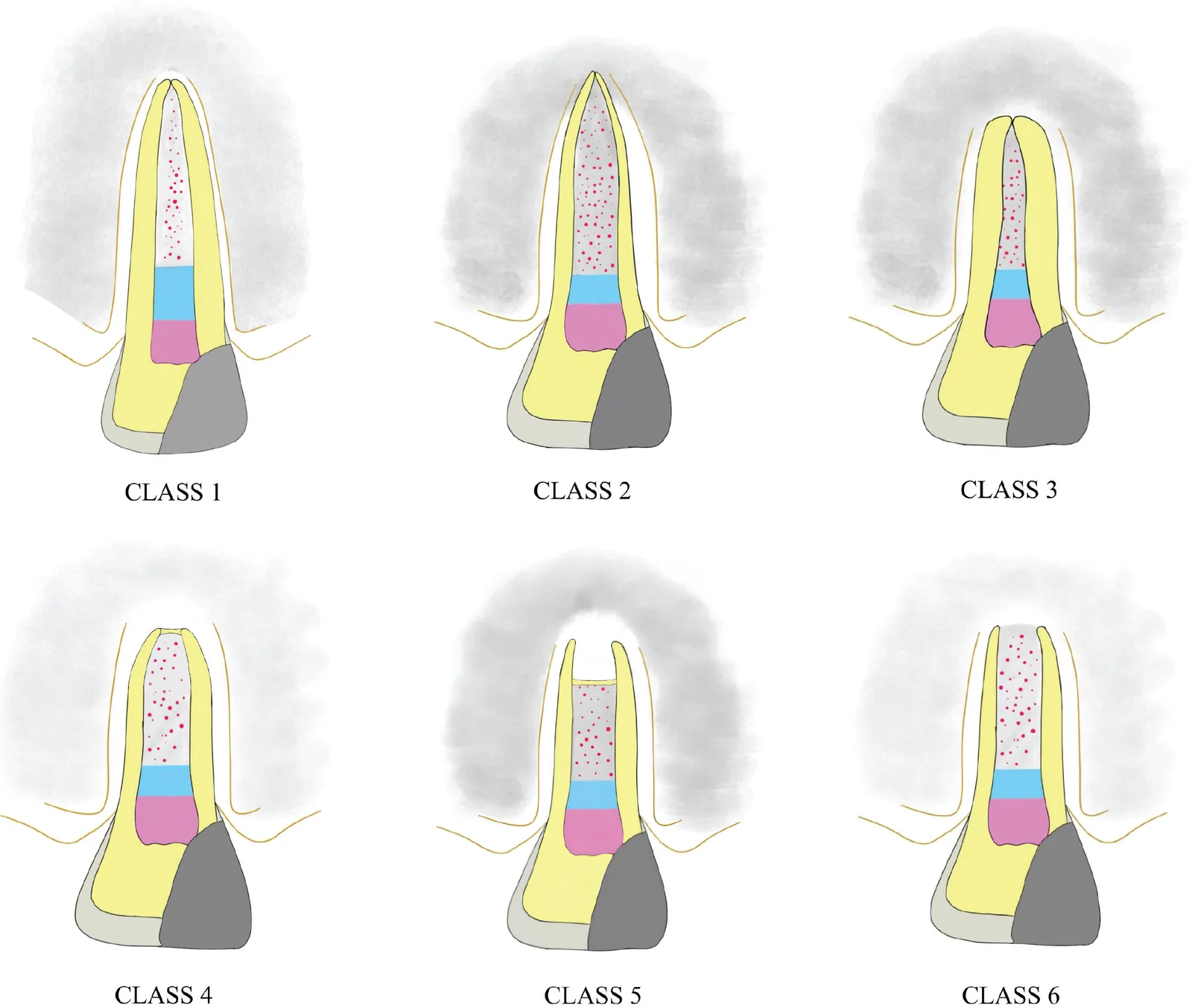
Illustrations depicting: Class 1- Radiographic apical closure with increase in both root length and dentin thickness, Class 2- Radiographic apical closure with increase in root length only, without any apparent change in dentin thickness, Class 3- Radiographic apical closure with increase in dentin thickness only, without any apparent change in root length, Class 4 – Radiographic apical closure without any increase in root length or dentin thickness, Class 5 – Radiographically visible barrier formed within the canals or coronal to root ends with no apparent change in root length or dentin thickness, Class 6 – No apparent change in root length, dentin thickness, or radiographic apical barrier but periapical healing is evident radiographically.

- **Class 1** – Radiographic apical closure with an increase in both root length and dentin thickness
- **Class 2** – Radiographic apical closure with an increase in root length only, without any apparent change in dentin thickness
- **Class 3** – Radiographic apical closure with an increase in dentin thickness only, without any apparent change in root length
- **Class 4** – Radiographic apical closure without any increase in root length or dentin thickness
- **Class 5** – Radiographically visible barrier formation within the canal or coronal to the root end, with no apparent change in root length or dentin thickness
- **Class 6** – No apparent change in root length, dentin thickness, or radiographic apical barrier, but radiographic evidence of periapical healing of a documented pretreatment periapical radiolucency.

Intracanal barrier formation, calcification, and periapical healing were additionally recorded. Each class can be further subdivided based on the presence or absence of intracanal calcification (partial or complete).

### Phase II – Application of proposed classification

The proposed classification framework was applied to an independent cohort of 54 cases treated between 2019 and 2023 to assess distribution and reproducibility of radiographic outcomes. Each case was independently assigned to one of the predefined classification categories based on follow-up radiographic findings by two blinded examiners who were trained and calibrated by a senior pedodontist. The assigned class represents the radiographic status at that observation point rather than an immutable biological endpoint; a tooth may move to another category if continued development becomes apparent during longer follow-up. Inter-examiner disagreements were resolved by consensus, and intra-examiner reliability was evaluated by repeat assessment of all the radiographs after a 15-day interval. Intra- and inter-examiner reliability were assessed using Cohen’s kappa coefficient. Any outcome not fitting the predefined categories was recorded as an alternative outcome category.

### Statistical analysis

The pilot cohort was used solely for development of the classification framework. Statistical analyses were performed exclusively for the subsequent cohort (Phase II) to evaluate the distribution and reproducibility of the proposed categories. Patient demographics, including tooth type, age, gender, and etiology for loss of vitality, and pre-treatment root development stage were reported in terms of frequencies and percentages. For analytical purposes, patients were categorized into two age groups: < 10 and ≥ 10 years. Associations of categorical variables, including age group and pre-treatment root developmental stage, with radiographic classification outcomes were assessed using Fisher’s exact test. A *p*-value < 0.05 was considered statistically significant.

## Results

Complete records of 44 patients (23 males and 21 females) aged 8–13 years were examined in Phase II of the present study, which included 54 teeth: 46 maxillary central incisors, 5 maxillary laterals, 1 mandibular central incisor, and 2 mandibular premolars. Subjects aged less than 10 years constituted 36/54 teeth (66.6%) of total cases, while 18/54 teeth (33.3%) belonged to the 10–13-year age group. Category assignment was based on the latest available follow-up radiograph for each tooth; the total available follow-up ranged from 24 to 53 months, with a minimum follow-up of 24 months. Only 2 teeth became non-vital due to caries, while the rest suffered trauma. Based on Cvek’s root development stage, 16 teeth belonged to stage 2, while 38 teeth belonged to stage 3 at the time of initiation of treatment. The main clinical protocol components, including irrigation regimen, intracanal medicament, and bleeding-induced scaffold, were largely consistent across cases; however, variations in interappointment interval, achieved bleeding level, and calcium-silicate barrier material were present.

Based on the radiographic evaluation of the cases at the last follow-up according to the proposed classification, cases were categorized into the following classes – Class 1 – 18 teeth, Class 2 – 3 teeth, Class 3 – 10 teeth, Class 4 – 9 teeth, Class 5 – 5 teeth, and Class 6 – 9 teeth (Figures 3 and 4). Although 24 months was selected as a long-term minimum to allow most readily detectable developmental changes to become apparent, later transition between categories remains possible. Class 1 occurred in 14/36 teeth (38.9%) from patients aged < 10 years and 4/18 teeth (22.2%) from those aged ≥ 10 years. No statistically significant association was observed between age group and radiographic classification outcome (Fisher’s exact test, *p* = 0.215). Similarly, pre-treatment root developmental stage showed no significant association with classification outcomes (Fisher’s exact test, *p* = 0.872) (Table 1). Class 1 occurred in 6/16 stage-2 teeth (37.5%) and 12/38 stage-3 teeth (31.6%). Partial intracanal calcification was seen in 19/54 teeth (35.18 %), while no cases of complete or total calcification were found. No other outcome class could be identified other than the six proposed classes. Given the relatively small sample size, these findings should be interpreted as preliminary, and larger studies are needed to further evaluate these associations. The inter-examiner Cohen’s kappa coefficient was 0.82, and the intra-examiner value was 0.86, indicating good inter- and intra-examiner agreement.

**Figure 3:**
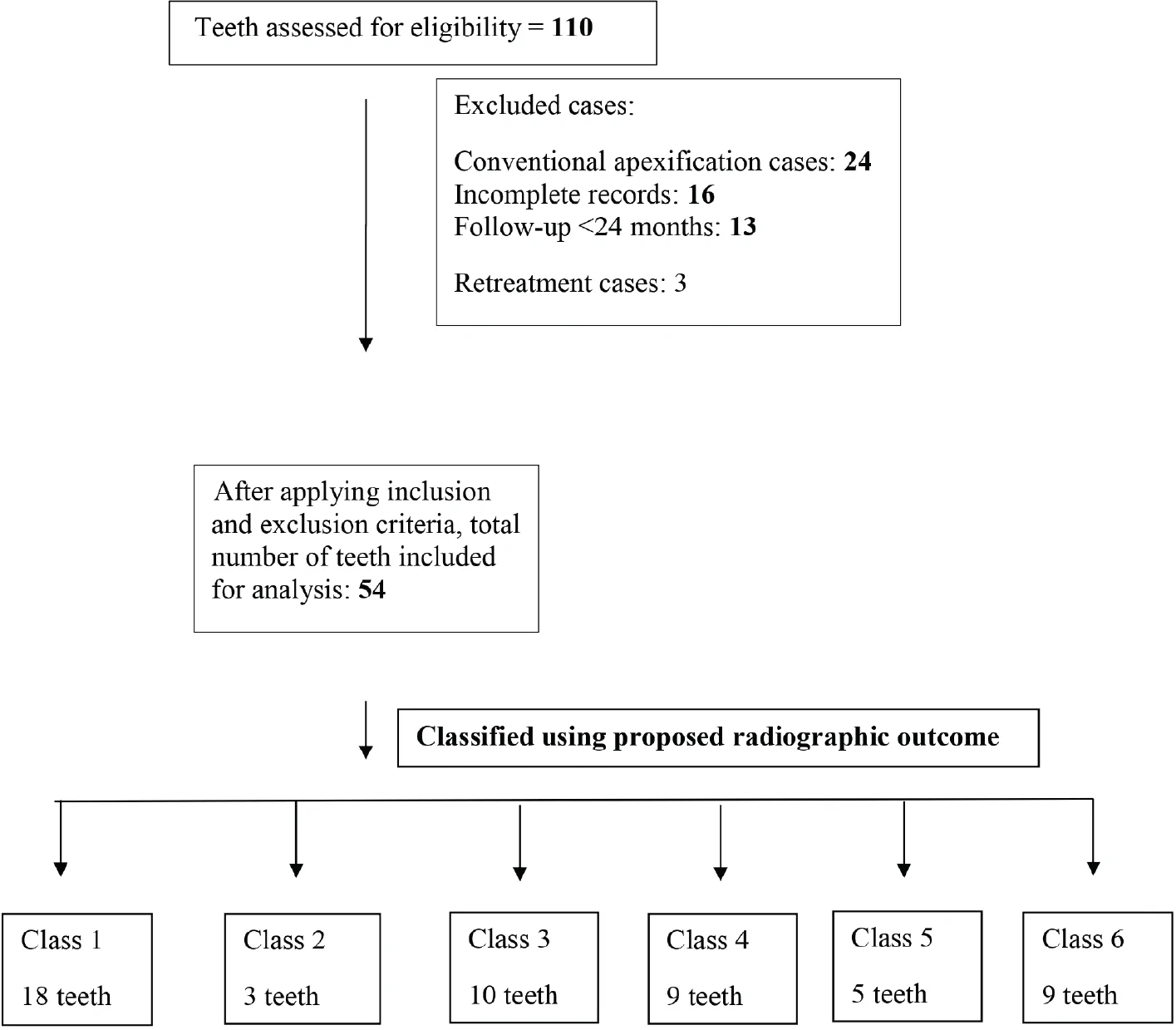
Flow chart depicting inclusion of cases.

**Figure 4:**
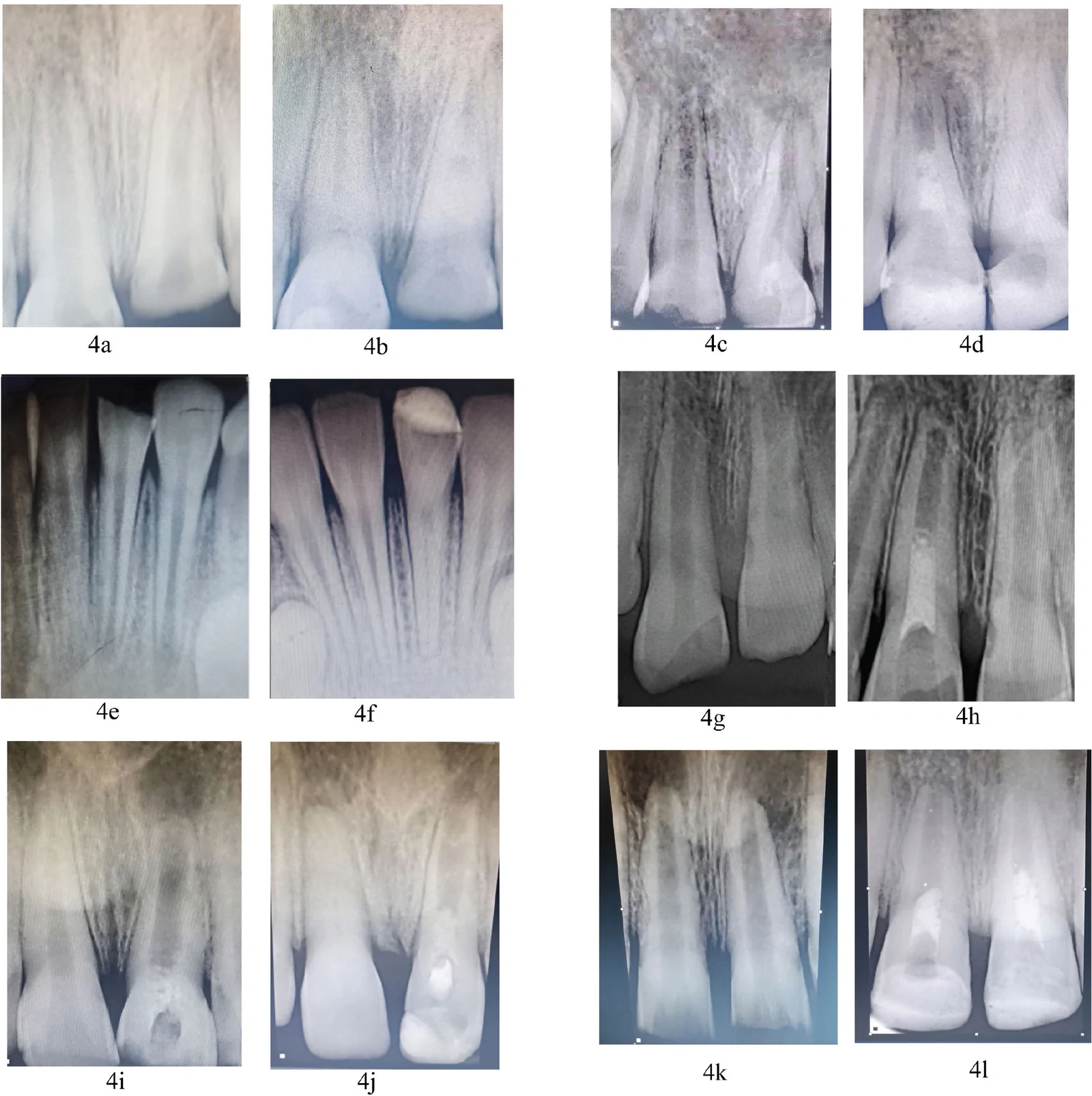
Pre- and post-treatment radiographs of six proposed classes: (a, c, e, g, I, and k) pre-treatment, (b) post-treatment class 1, (d) post-treatment class 2, (f) post-treatment class 3, (h) post-treatment class 4, (j) post-treatment class 5, (l) post-treatment class 6.

**Table 1:** Distribution of cases according to age and pretreatment root development stage across radiographic outcome classes.

Variable	Category	Class 1 (*n* = 18)	Class 2 (*n* = 3)	Class 3 (*n* = 10)	Class 4 (*n* = 9)	Class 5 (*n* = 5)	Class 6 (*n* = 9)	*p* †
Age	< 10 years (*n* = 36)	14	3	8	4	2	5	0.215
	≥ 10 years (*n* = 18)	4	0	2	5	3	4	
Pretreatment root development stage	Cvek’s stage 2 (*n* = 16)	6	1	2	2	1	4	0.872
	Cvek’s stage 3 (*n* = 38)	12	2	8	7	4	5	

† Fisher’s exact test.

Pretreatment periapical radiolucency was present in 14/54 teeth (25.9%). Of these 14 cases, nine were classified as Class 6, two each as Class 3 and Class 4, and one as Class 5 at radiographic follow-up. Complete radiographic resolution of the periapical radiolucency was observed in 11/14 lesions (78.6%), including nine Class 6 and two Class 3 cases. The remaining three lesions demonstrated clear radiographic reduction without progression and were considered to show ongoing healing. All nine Class 6 cases with documented pretreatment periapical radiolucency demonstrated complete radiographic resolution at follow-up.

## Discussion

The clinical success of REPs has traditionally been defined by the resolution of symptoms and periapical healing. However, radiographic signs such as apical closure, root lengthening, and dentinal wall thickening provide critical insights into the biological success and long-term prognosis of the treated tooth. Radiographic evaluation remains the mainstay for assessing the outcomes of REPs, especially given the ethical and practical limitations of histologic assessment in human studies.

In this study, a structured radiographic classification system was proposed and applied to support consistent categorization of radiographic outcomes and provide a more structured description of successful healing following REPs. This system stratifies radiographic outcomes into six main types, ranging from complete healing with root maturation (Class 1) to no evident radiographic changes (Class 6). By evaluating apical closure, root lengthening, dentinal wall thickening, and periapical healing, this framework provides structured assessment of individual regenerative outcomes. Moreover, the inclusion of optional subcategories such as canal obliteration or calcific tissue formation adds further granularity for both clinical and research purposes.

The distribution of radiographic outcomes observed in this study can be interpreted in the context of several biological and procedural factors reported in the literature, as described by Chen et al. [22] and subsequent authors. Chen et al. [22] described five qualitative radiographic response patterns following revascularization, encompassing root maturation, apical closure, continued development with an open apex, canal obliteration, and intracanal barrier formation. Thakkar et al. [24] subsequently applied the Chen response categories to compare blood-clot and platelet-rich fibrin (PRF) scaffolds and found no significant difference in apical response between the groups. Shivashankar et al. [23] combined the Chen categories with Schei’s ruler for separate assessment of root lengthening and dentinal-wall thickening and the Periapical Index for periapical healing. More recently, Erdoğan et al. [25] evaluated radiographic root area together with apical closure, hard-tissue formation, intracanal calcification, and external root resorption. These studies demonstrate variation in the methods used to assess post-REP radiographic outcomes. The present framework does not replace these approaches but modifies and extends the Chen categories by separately classifying combinations of apical closure, root lengthening, and dentinal-wall thickening while recording additional barrier, calcification, and periapical-healing patterns. The conceptual relationship between the Chen categories and the present framework is summarized in Table 2, while the methodological differences between the Chen et al. study and the present study are presented in Table 3.

**Table 2:** Comparison of the Chen et al. [22] radiographic response categories with the proposed framework.

Chen’s classification	Proposed new classification (Singhal and Bansal classification system)	Relationship and distinction of proposed classes with Chen’s type
Type 1: Increased thickening of the canal walls and continued root maturation	**Class 1** – Radiographic apical closure with an increase in both root length and dentin thickness	Resembles Chen’s Type 1: wall thickening + continued root maturation (The present class explicitly requires the three components to occur together)
Type 2: No significant continuation of root development with root apex becoming blunt and closed	**Class 2** – Radiographic apical closure with an increase in root length only, without any apparent change in dentin thickness	Resembles Part of Chen’s Type 1 (The present system separates lengthening and apical closure from wall thickening.)
Type 3: Continued root development with the apical foramen remaining open	**Class 3** – Radiographic apical closure with an increase in dentin thickness only, without any apparent change in root length	Resembles Part of Chen’s Type 1 (The present system isolates thickening and apical closure without appreciable lengthening as a separate pattern.)
Type 4: Severe calcification (Obliteration) of the canal space	**Class 4** – Radiographic apical closure without any increase in root length or dentin thickness	Resembles Chen’s Type 2: no significant continued development; blunt/closed apex (The present class explicitly states absence of changes in two dimensions.)
Type 5: A hard tissue barrier formed in the canal between the coronal MTA plug and the root apex	**Class 5** – Radiographically visible barrier formation within the canal or coronal to the root end, with no apparent change in root length or dentin thickness	Resembles Chen’s Type 5: hard-tissue barrier between MTA and apex (Substantial overlap; the present class additionally specifies absence of dimensional root change.)
	**Class 6** – No apparent change in root length, dentin thickness, or radiographic apical barrier, but radiographic evidence of periapical healing of a documented pretreatment periapical radiolucency	No direct Chen type (Potential extension, but ‘healing’ is applicable only to a documented pretreatment lesion.)
	Calcification subdivision	Type 4: severe canal calcification/obliteration (Chen treated obliteration as a principal type; the present framework treats partial/complete calcification as a modifier that may coexist with another class.)

MTA: mineral trioxide aggregate.

**Table 3:** Methodological comparison of the Chen et al. [22] classification and the present study.

Domain	Chen et al.	Present study	Interpretation
Design	Descriptive case series; one cohort	Two-phase retrospective study: development cohort followed by an independent temporal application cohort	More structured internal application, but not external validation.
Sample	20 teeth/20 patients	Phase I: 20 cases; Phase II: 54 teeth/44 patients	Larger application cohort; clustering requires consideration.
Baseline disease	All teeth had apical periodontitis/abscess	Necrotic teeth with or without periapical pathology; 14/54 had a baseline radiolucency	Different disease spectrum; Class 6 healing must be limited to baseline lesions.
Main etiology/teeth	10 incisors and 10 premolars; trauma, caries, or dens evaginatus	Predominantly incisors and trauma (52/54 trauma; 2/54 caries)	Present findings mainly reflect traumatized anterior teeth.
Follow-up	6–26 months; schedule not controlled	Primary classification stated at 24 months; total available follow-up 24–53 months	Longer minimum follow-up of 24 months, that allow appreciable changes in root maturation.
Radiographic assessment	Primarily qualitative response descriptions	Defined CEJ/apex and 6.5-mm measurement landmarks	More explicit landmarks, although quantitative thresholds and image correction were absent.
Reliability/statistics	No examiner-reliability analysis or inferential testing	Blinded examiners; Cohen kappa; exploratory Fisher tests	Methodological addition of the present study.

CEJ: cemento-enamel junction

Patient age is a well-established determinant of regenerative success, with younger individuals demonstrating more robust responses due to higher stem cell activity and a more favorable apical microenvironment. The proliferative and differentiation potential of mesenchymal stem cells decreases with age [29, 30]. Several authors have demonstrated significantly greater root development in younger patients (< 12 years old) compared to those older than 12 years. Moreover, most patients with immature necrotic permanent teeth in previous studies were below 17 years of age, making them ideal candidates for REPs [20]. In the present study, a numerically higher proportion of cases categorized as Class 1 (radiographic apical closure with an increase in both root length and dentin thickness) – were observed in children younger than 10 years of age; however, this difference did not reach statistical significance (*p* = 0.215), and the trend should therefore be interpreted with caution.

The stage of root development at the time of treatment is a critical determinant of regenerative outcomes. Teeth exhibiting wide-open apices and shorter roots are more conducive to continued development, as they facilitate easier access for stem cell homing and vascular ingrowth. A study by Theekakul et al. [21] reported that teeth at stage 3 of root development, as per Moorrees’ classification [31], demonstrated a significant increase in root length and width compared to those at stages 4 or 5. Similarly, teeth with preoperative canal diameters of ≥ 1 mm showed greater increase in root length, thickness, and apical narrowing [20]. However, in the present study, no significant association between pre-treatment root developmental stage and radiographic outcome classes was observed, possibly due to the inclusion of cases limited to Cvek’s stages 2 and 3 at the initiation of treatment. To draw definitive conclusions regarding treatment outcomes across various stages of root development, future prospective long-term studies encompassing a larger cohort with diverse developmental stages are warranted.

The most favorable radiographic outcome, classified as Class 1 in the proposed system, encompasses an increase in root length and dentin thickness, along with a decrease in apical diameter. In the present study, this outcome was the most prevalent, observed in 18 out of 54 teeth (33.3%) following REPs. An increase in root length was noted in both Class 1 and Class 2 outcomes, totaling 21 cases (38.9%), while an increase in dentin thickness was observed in Class 1 and Class 3 outcomes, accounting for 28 cases (51.85%). However, due to the retrospective nature of the study, with a limited number of patients and the diverse distribution of cases across categories, definitive correlations with all pre-treatment variables could not be established. Pulp necrosis due to trauma or caries influences regenerative outcomes. Previous studies report better survival of Hertwig’s epithelial root sheath and apical papilla in carious lesions than traumatic injuries [32–34]. Thus, traumatized teeth are less likely to complete root development after REPs [35]. In the present study, only 2 cases of immature roots due to caries were included; the rest were attributed to trauma, limiting comparisons based on etiology.

Presence of periapical radiolucency was seen in 14 cases. Among the 14 teeth with pretreatment periapical radiolucency, complete radiographic resolution was observed in 11 teeth (78.6%). Nine of these 11 teeth (81.8%) were classified as Class 6, while the remaining two were classified as Class 3. Three teeth demonstrated clear radiographic reduction without complete resolution and were considered to show ongoing healing. This could be attributed to the size and chronicity of the periapical lesion, as larger or long-standing radiolucencies may reduce the likelihood of rapid or complete healing due to higher concentrations of residual bacteria [36].

Procedural factors such as disinfection protocol, intracanal medicament, and scaffold type have been reported in the literature to influence radiographic outcomes in REPs. These variables were not independently examined in the present study, as the clinical protocol was largely standardized across all cases. Sodium hypochlorite (NaOCl) is widely used for its antimicrobial and tissue-dissolving properties, but higher concentrations are cytotoxic to stem cells of the apical papilla [37–39]. EDTA (17%) mitigates this effect by improving stem cell viability and growth factor release [39]. Although the AAE recommends 1.5–3% NaOCl, a systematic review reported that low concentrations (≤ 2%) provide comparable disinfection with better clinical and radiographic outcomes due to reduced cytotoxicity [40]. Accordingly, 1.5% NaOCl was used in the present study to balance antimicrobial efficacy with preservation of the regenerative microenvironment. Calcium hydroxide promotes stem cell differentiation but may increase intracanal calcification [35]. Therefore, it was not used in this study. TAP provides effective disinfection and may promote greater dentinal thickening, whereas calcium hydroxide favors apical closure [41]. The lower incidence of intracanal calcification observed in the present study may be related to the use of TAP; however, previous studies report no prognostic effect of intracanal calcification [35]. PRP as an intracanal scaffold may improve periapical healing, but no scaffold has been shown to significantly outperform others in root maturation [42]. Induced bleeding is preferred due to its simplicity and was exclusively used in this study. Cases with insufficient bleeding or alternative scaffolds (e.g. PRF, PRP) were excluded to reduce variability.

The duration of radiographic follow-up influences interpretation of outcomes in REPs. Root formation is typically detectable after a mean of 16 months post-REP [35, 43]. Jiang et al. [35] reported that root canal calcification may increase during long-term follow-up, although it does not adversely affect tooth prognosis. Similarly, Linsuwanont et al. [44] observed that conventional 2D radiographs may suggest complete root maturation, whereas cone-beam computed tomography (CBCT) imaging may reveal incomplete or abnormal development. This discrepancy questions the reliability of prior success assessments.

In a study by Mittmann et al. [45], outcomes of regenerative endodontics in traumatized immature teeth with luxation or avulsion were evaluated. The authors reported a significant reduction in apical diameter but no improvement in root length or dentin thickness, concluding that revascularization offered no advantage over apexification in such cases, likely due to greater damage to the periodontal ligament and supporting structures. In contrast, the present study included only subluxation injuries or dental hard tissue injuries (Ellis Class 3 and 4), which likely preserved the periodontal ligament and Hertwig’s epithelial root sheath, enabling continued root development. Consequently, positive changes in root length, dentin thickness, and apical diameter were observed.

By integrating these variables into a structured classification system, clinicians and researchers can improve consistency in outcome reporting and identify potential predictors of success or failure. The classification should be interpreted specifically as a framework for categorizing radiographic patterns observed following clinically successful REPs rather than as a comprehensive classification of all possible REP outcomes. This system may aid in patient counseling, risk stratification, and optimization of clinical protocols. While high inter- and intra-examiner reliability indicates reproducibility, future multicenter, prospective studies should evaluate whether specific classes correlate with long-term clinical outcomes, tooth survival, or need for further intervention.

## Limitations

This retrospective study relied on existing clinical and radiographic records, which may contain inconsistencies in imaging techniques, documentation, and follow-up intervals. No dedicated software-based image calibration or correction for radiographic projection errors was performed. Consequently, minor variations in radiographic angulation and magnification may have influenced the assessment of root dimensions and radiographic outcome classification. The absence of CBCT in all cases may also have affected the accuracy of root and periapical assessments.

The fixed 6.5-mm CEJ-based measurement point does not necessarily represent an identical proportional root location across teeth with different baseline root lengths. This landmark was selected as a pragmatic and reproducible reference for the predominantly maxillary central incisor cohort. Future prospective studies may consider normalization of the measurement site, such as assessment at approximately 50% of baseline root length.

Inter-rater reliability was assessed using Cohen’s κ as the available reliability measure; additional agreement metrics, such as percentage agreement, confidence intervals, or specific disagreement categories, were not available and therefore could not be retrospectively calculated.

The assigned radiographic category reflects the status of the tooth at the latest available follow-up assessment and may not represent its final developmental outcome. Continued root development with a persistently open apex may represent a transitional rather than a final response and may be encountered more frequently with shorter follow-up periods. Although most readily detectable developmental changes were expected to have occurred by 24 months, further root development, partial apical narrowing, or barrier formation combined with root maturation cannot be excluded and may result in reclassification. Assessment of continued healing was based on comparative evaluation of serial radiographs and was not based on a validated periapical scoring system or predefined percentage reduction.

Therefore, the proposed categories should be interpreted at a clearly specified follow-up point, with long-term serial assessment required to determine their stability and possible transitions. Further classes or modifiers may be incorporated as larger, more heterogeneous cohorts reveal additional radiographic patterns.

The six-class framework should not be considered an exhaustive taxonomy of all possible radiographic outcomes following REPs. The proposed framework is intended only to categorize radiographic outcomes; it is not a clinical decision algorithm and does not direct treatment selection. This study evaluates internal applicability and reproducibility only and does not establish external validity, comprehensiveness, or superiority over the Chen classification.

## Strengths

Despite its limitations, this study has several strengths. It is among the first to propose and implement a structured radiographic classification system tailored to REPs. The study was conducted using a two-phase design, wherein the classification framework was developed using a pilot cohort and subsequently applied and assessed in an independent cohort of patients. The independent application cohort provided an opportunity to examine the internal applicability and reproducibility of the proposed framework, distinguishing degrees of response rather than relying on binary outcomes. The study attempted to minimize major procedural variability through a largely consistent treatment protocol and predefined eligibility criteria. A reasonably large and diverse sample further enhances the validity and applicability of the findings. Collectively, these strengths provide a foundation for future studies, protocol optimization, and refinement of outcome measures in regenerative endodontics.

## Conclusion

- This study proposes a structured modification and extension of existing radiographic response patterns following REPs, addressing the lack of uniform outcome categorization.
- By separately categorizing apical closure, root lengthening, and dentinal-wall thickening, together with barrier formation, calcification, and periapical healing, the framework provides a clearer and more systematic assessment of radiographic outcomes.
- The framework demonstrated strong internal reproducibility and offers a practical basis for consistent clinical and research reporting. Independent multicenter validation would further establish its broader applicability.
- Future prospective studies using standardized imaging and longer follow-up should examine associations between the proposed classes and biological, clinical, and procedural factors and integrate radiographic findings with clinical and patient-reported outcomes.

## Data Availability

Data sharing will be available upon reasonable request from the corresponding author.
